# A 3D Multilevel Heterostructure Containing 2D Vertically Aligned MoS_2_ Nanosheets and 1D Sandwich C-MoS_2_-C Nanotubes to Enhance the Storage of Li^+^ Ions

**DOI:** 10.3390/nano13142088

**Published:** 2023-07-18

**Authors:** Yiyang Zhao, Wenhao Luo, Huiqing Luo, Xiaodi Liu, Wenjun Zheng

**Affiliations:** 1 College of Chemistry and Pharmaceutical Engineering, Nanyang Normal University, Nanyang 473061, China; 2 Key Laboratory of Advanced Energy Materials Chemistry (MOE), Department of Chemistry, College of Chemistry, Nankai University, Tianjin 300071, China

**Keywords:** MoS_2_, nanotubes, nanosheets, sandwich heterostructure, lithium-ion batteries

## Abstract

To overcome the disadvantages of the MoS_2_ anode for LIBs in terms of low intrinsic conductivity, poor mechanical stability, and adverse reaction with electrolytes, a 3D multilevel heterostructure (VANS-MoS_2_-CNTs) has been successfully prepared by a simple hydrothermal method followed by thermal treatment. VANS-MoS_2_-CNTs are made up of 2D vertically aligned MoS_2_ nanosheets (VANS) and 1D sandwich C-MoS_2_-C nanotubes (CNTs). The sandwich-like nanotube is the core part, which is made up of the MoS_2_ nanotube covered by carbon layers on both side surfaces. Due to the special heterostructure, VANS-MoS_2_-CNTs have good conductivity, high structured stability, and excellent Li^+^/electron transport, resulting in high discharge capacity (1587 mAh/g at a current density of 0.1 A/g), excellent rate capacity (1330 and 730 mAh/g at current densities of 0.1 and 2 A/g, respectively), and good cyclic stability (1270 mAh/g at 0.1 A/g after 100 cycles).

## 1. Introduction

As is well known, the low specific capacity of graphite limits the utilization of lithium-ion batteries (LIBs) in some large-capacity energy storage devices such as electric vehicles and hybrid electric vehicles [1]. To address this issue, the exploration of new and effective anodic materials with high capacity and good cyclic stability is vitally important for future applications of LIBs [2,3,4].

Recently, layer-structured MoS_2_ has received much attention in several fields, such as photocatalysis [5,6], supercapacitors [7,8], batteries [9,10], photoelectrochemical water splitting for hydrogen production [11,12,13], solid lubricants [14], etc. Among various anodic materials, MoS_2_ is one of the most promising candidates because of its high theoretical capacity (approximately 670 mAh g^−1^) and unique structure [15,16,17]. Therefore, some MoS_2_ nanomaterials, involving nanospheres [18], nanotubes [19,20], hollow nanoparticles [21], and nanoflakes [22], have been fabricated as anodic materials for large-capacity LIBs. However, MoS_2_ shows drawbacks in low intrinsic conductivity, poor mechanical stability, and adverse reactions with electrolytes, leading to an inferior rate capability and fast capacity decay [23]. Hence, it is highly expected that the structure of MoS_2_ is designed to enhance the storage of Li^+^ ions.

To address these problems, MoS_2_ anodic material has been mainly designed in the following two ways. The first way is to prepare 2D single-layer MoS_2_ nanostructures. With the number of layers decreasing to a single layer, the crystal structure of MoS_2_ transforms from the 2H semiconductor phase to the 1T metallic phase [24]. The 1T metallic phase of MoS_2_ shows much higher electronic conductivity than the 2H semiconductor phase [25,26]. However, as the 1T-MoS_2_ electrode is exposed to the electrolyte, the occurrence of adverse reactions cannot be avoided. The second way is to find suitable supporters (that is, TiO_2_, graphene, and carbon materials) to obtain MoS_2_-based composite materials [27,28,29,30,31,32,33,34]. The supporters can provide feasible electron transport pathways; therefore, the second way is an effective strategy. For example, Lou et al. employed mesoporous carbon (CMK-3) as supporter and prepared CMK-3/MoS_2_ composites, which deliver a reversible capacity of 934 mAh/g at 0.4 A/g after 150 cycles [35]. In addition to improving the conductivity by supporters, sandwich MoS_2_/carbon heterostructures have been tailored to simultaneously improve the stability of the electrode [36,37,38,39]. For instance, Fang et al. have synthesized a C/MoS_2_/C trilayer nanostructure, in which the MoS_2_ monolayer was fully covered by the mesoporous carbon layers [39]. The layer-by-layer heterostructure shows a high discharge capacity of about 1400 mAh/g at a current rate of 100 mA/g after 300 cycles. The sandwich structure of MoS_2_/carbon possesses the advantages of stable structure and rapid electron transport; however, the insertion and extraction of Li^+^ ions into and from the electrode are hampered by the coated carbon layers. Considering the advantages of the 2D nanostructures and sandwich-like structures of MoS_2_/carbon composite materials, it is very meaningful to design and synthesize a 3D multilevel heterostructure to enhance the electrochemical performance of MoS_2_.

Herein, a novel MoS_2_ heterostructure (VANS-MoS_2_-CNT) has been synthesized by a facile, low-cost, and green route using glucose as the carbon source, which is beneficial for practical applications. VANS-MoS_2_-CNTs are composed of 2D vertically aligned MoS_2_ nanosheets (VANS) and 1D sandwich-like C-MoS_2_-C nanotubes (CNTs). The nanotubular structure supplies abundant paths for the rapid transport of Li^+^; moreover, carbon layers are coated on the inner and outer surfaces of MoS_2_ nanotubes, effectively preventing the adverse reaction between MoS_2_ and the electrolyte. VANS are the extension part of the MoS_2_ nanotubes and they are not covered by carbon layers; therefore, they can still maintain the advantage of 2D nanostructures. Due to their special structure, the obtained VANS-MoS_2_-CNT electrode displays high capacity, good rate capacity, and excellent cyclic stability. This work could provide some ideas for the rational structural design of MoS_2_ to acquire superior performance in the storage of Li^+^ ions.

## 2. Materials and Methods

### 2.1. Fabrication of VANS-MoS_2_-CNTs

All chemicals were analytical reagent grade without additional purification and were obtained from Saan Chemical Technology Co., Ltd. (Shanghai, China). Firstly, the VANS-MoS_2_-nanotubes (VANS-MoS_2_-NTs) template was fabricated based on our previous work [40]. An amount of 0.025 g of VANS-MoS_2_-NTs and 0.15 g of glucose were added to deionized water (15 mL) and stirred for 3 h until the glucose was uniformly adsorbed on the surface of the template. The as-obtained precursor was calcinated at 800 °C for 5 h under an atmosphere of argon.

### 2.2. Materials Characterization

The crystalline structure of the sample was characterized using a Bruker D8 FOCUS X-ray powder diffractometer (XRD, Cu *K*_α_ radiation, Bruker Corporation, Billerica, MA, USA). The size, shape, and nanostructure were investigated on a ZEISS MERLIN Compact scanning electron microscope (SEM, Carl Zeiss AG, Oberkochen, Germany) and a Tecnai G2 F20 transmission electron microscope (TEM, Frequency Electronics Inc., Hillsboro, OH, USA). X-ray photoelectron spectra (XPS) were obtained using a Thermo ESCALAB 250XI electron spectrometer (Thermo Fisher Scientific Inc., Waltham, MA, USA). Raman spectrum was taken by a Renishaw inVia Raman microscope (Renishaw Company, Gloucestershire, UK). Nitrogen adsorption/desorption test was performed on an ASAP 2020/Tristar 3000 instrument (Micromeritics Instrument Corporation, Norcross, GA, USA). The amount of carbon materials in the sample was assessed using SDT Q600 thermal gravimetric analysis (TG, TA Instruments, New Castle, DE, USA).

### 2.3. Electrochemical Measurements

The as-prepared VANS-MoS_2_-CNTs, acetylene black, and binder in a weight ratio of 80:10:10 were mixed and then uniformly coated on Cu foil. Lithium metal was used as the counter and reference electrode. LiPF_6_ (1 mol/L) dissolved in ethylene carbonate, ethylene methyl carbonate, and dimethyl carbonate (1:1:1, *v*/*v*/*v*) was the electrolyte. The cell was assembled in an argon-filled glovebox, where H_2_O and O_2_ concentrations were lower than 5 ppm. Cyclic voltammetry (CV) and electrochemical impedance spectroscopy (EIS) were tested on a CHI660B electrochemical workstation (Shanghai Chenhua Instrument Ltd., Shanghai, China). Galvanostatic charge/discharge measurements were performed on a Land CT2001 automatic battery tester (Wuhan Shenglan Electronic Technology Co., Ltd., Wuhan, China).

## 3. Results and Discussions

### 3.1. Structural Characterization

**Figure 1:**
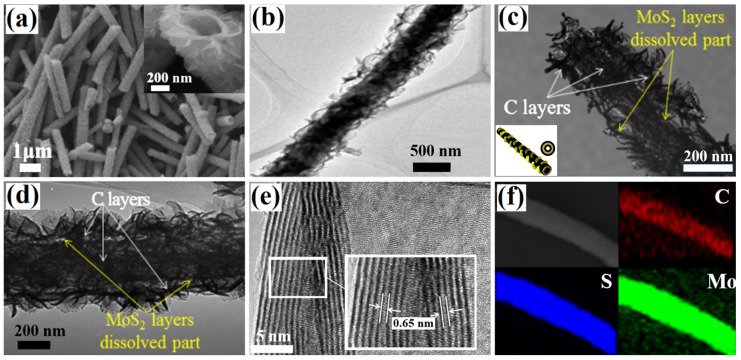
(**a**) SEM, (**b**) TEM, (**e**) HRTEM, and (**f**) EDS mapping images of VANS-MoS_2_-CNTs. TEM images taken on the (**c**) top and (**d**) middle of a typical composite nanotube after acid treatment, and the inset of (**c**) is the schematic illustration of VANS-MoS_2_-CNTs.

As shown in the SEM image of the as-obtained VANS-MoS_2_-NT template (Figure S1a), it has a well-dispersed tubular structure with 400–500 nm in width. When the VANS-MoS_2_-NT template is converted to a VANS-MoS_2_-CNT, the tubular structure is inherited (Figure 1a). Carbon layers are formed on the internal and external surfaces of the tubular template; therefore, the as-obtained VANS-MoS_2_-CNTs have different diameters from the template. The high-magnification SEM images (insets of Figure S1a and Figure 1a) verify the differences in the nanotube mouths between the VANS-MoS_2_-CNTs and the VANS-MoS_2_-NTs. Specifically, the internal diameter of the VANS-MoS_2_-NTs is ca. 400 nm, while it becomes ca. 300 nm for the VANS-MoS_2_-CNTs. This change may be caused by the generation of a carbon layer on the inner wall of VANS-MoS_2_-CNTs, which can be proved by the TEM image of VANS-MoS_2_-CNTs.

Carbon is coated on the inner/outer surfaces of the VANS-MoS_2_-NTs and consequently a sandwich structure is formed, which makes the nanotubes of the VANS-MoS_2_-CNTs dark in the TEM image. Hence, as can be seen in Figure 1b, the hollow tubular structure of the VANS-MoS_2_-CNTs is not so obvious. To further prove the sandwich structure, the MoS_2_ layer is removed by acid treatment and the product is further tested by TEM. TEM images taken on the top part (Figure 1c) and middle part (Figure 1d) of a typical nanotube provide the same information. That is, as illustrated in the inset of Figure 1c, the MoS_2_ layer (represented by a dark yellow circle) is dissolved and a vacant space is formed between the two layers of carbon (represented by black circles), which is evidence for the sandwich-like structure. The nanostructures of VANS in VANS-MoS_2_-CNTs are researched using HRTEM (Figure 1e). The *d*-spacing in the MoS_2_ nanosheets is ca. 0.65 nm, consistent with the lattice fringe distance of the (002) plane of 2H-MoS_2_. Moreover, the Mo, S, and C elements are uniformly distributed in VANS-MoS_2_-CNTs (Figure 1f). The above results confirm that 1D C-MoS_2_-C sandwich nanotubes with free-standing MoS_2_ nanosheets have been prepared, and the 3D composite nanostructures not only increase the conductivity, but also improve the structural stability of the sample. In addition, VANS-MoS_2_-CNTs have a large specific surface area of ca. 57.2 m^2^/g for the unique heterostructure (Figure S2), which is conducive to improving the electrochemical properties [41].

**Figure 2:**
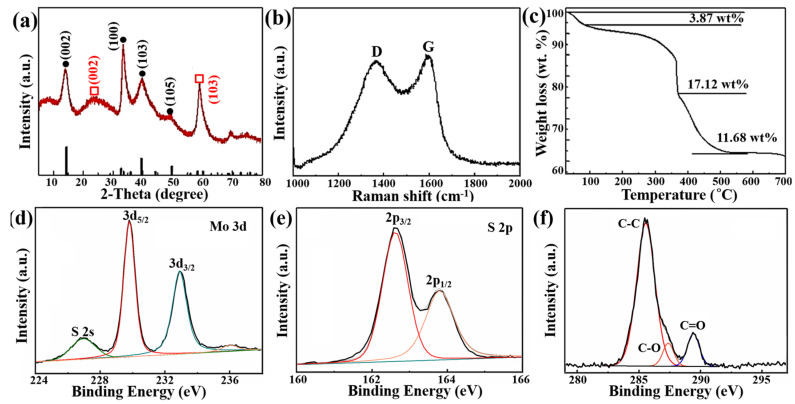
(**a**) XRD pattern, (**b**) Raman spectrum, (**c**) TG curve, and (**d**–**f**) Mo 3d, S 2p, and C 1s XPS spectra of VANS-MoS_2_-CNTs. D and G in (**b**) represent the Raman D and G bands. The colored lines in (**d**–**f**) represent the fitted results of different electron orbits and bonds.

XRD, Raman, TG, and XPS tests were performed to further confirm the crystal structure and composition of the VANS-MoS_2_-CNTs. Figure 2a shows the XRD pattern of the VANS-MoS_2_-CNTs. All diffraction peaks can be indexed to 2H-MoS_2_. The peaks at 14.4°, 32.7°, 39.5°, 49.8°, and 70.1° can be ascribed to the (002), (100), (103), (105), and (108) reflection planes of 2H-MoS_2_, respectively (JCPDS No. 73-1508). Moreover, the (110) plane of 2H-MoS_2_ at 58.3° is hard to observe due to the emergence of a strong peak of a 2H-graphite (103) plane at 59.7° (JCPDS No. 41-1487). The other peak at 26.3° can be attributed to the (002) facet of 2H-graphite. The XRD result indicates that VANS-MoS_2_-CNTs have good crystallinity and the carbon layers exist as graphite. The degree of graphitization of carbon material is usually characterized by XRD and Raman techniques [42,43]; therefore, the Raman spectrum of VANS-MoS_2_-CNTs was also obtained. As shown in Figure 2b, two peaks located at 1349 and 1583 cm^−1^ can be assigned to the D and G bands, respectively. It is obvious that the intensity of the G band is higher than that of the D band, suggesting that the carbon layers have a high degree of graphitization [44,45]. The graphite content in the VANS-MoS_2_-CNTs was further measured by a TG test. In the TG curve (Figure 2c), the first weight loss (3.87 wt%) occurs below 110 °C, which is attributed to the desorption of a small amount of water in the composites. The second weight loss (17.12 wt%) between 300 and 380 °C is attributed to the oxidation of MoS_2_ to MoO_3_. The final weight loss (11.68 wt%) from 384 to 500 °C is inferred to result from the decomposition/burning of graphitized carbon in VANS-MoS_2_-CNTs [46]. Thus, the content of graphitized carbon in VANS-MoS_2_-CNTs is about 11.68 wt%.

XPS spectra are used to analyze the chemical state of the VANS-MoS_2_-CNTs (Figure 2d–f). As shown in Figure 2d, the binding energies at around 232.9 and 229.8 eV are attributed to the Mo 3d_3/2_ and Mo 3d_5/2_ orbitals of Mo^4+^, respectively; furthermore, the peak located at 226.9 eV belongs to S 2s [47,48]. The small peak at 236.1 eV is assigned to Mo^6+^ 3d_3/2_, which may be due to the oxidation of Mo^4+^ on the surface of MoS_2_ [49,50]. The two peaks at 163.8 and 162.6 eV can be attributed to S^2−^ of MoS_2_ (Figure 2e) [51]. The characteristic peaks of C=O, C-O, and C-C located at 289.7, 287.2, and 285.2 eV can be seen in the C 1s spectrum shown in Figure 2f, which is consistent with the results for graphite [52,53]. 

### 3.2. Formation Mechanism

**Figure 3:**
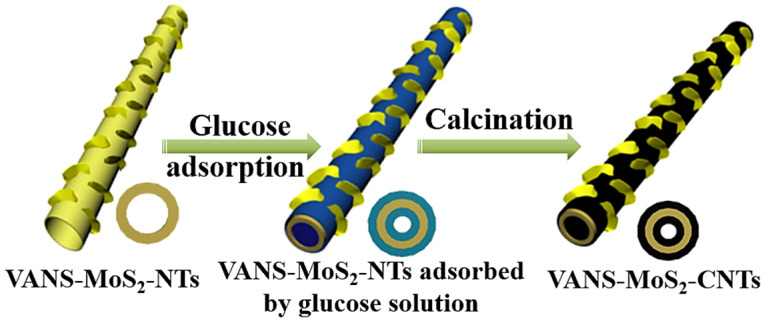
Schematic illustration of the formation mechanism of VANS-MoS_2_-CNTs.

Due to the special heterostructures of VANS-MoS_2_-CNTs, it is meaningful to discuss their formation mechanism. Firstly, based on our previous work [40], the VANS-MoS_2_-NTs template was prepared with the assistance of 1-n-butyl-3-methyl-imidazolium thiocyanate ([BMIM]SCN). The glucose solution was then employed as the carbon source to obtain sandwich-like C-MoS_2_-C heterostructures. The formation mechanism of VANS-MoS_2_-CNTs can be described by the following steps shown in Figure 3. During the calcination process, the concentration of the glucose solution is the vital parameter to keep VANS uncovered by carbon. As shown in Figure S3, as the glucose solution concentration is 20 g/L, the MoS_2_ nanotubes are covered by thick and uniform carbon layers, and the signal of the C element is wider than that of the Mo and S elements. When the concentration of the glucose solution is 10 g/L, the MoS_2_ nanosheets are clearly observed in Figure 1b. That is to say, the MoS_2_ nanosheets are exposed on the outer surfaces of C-MoS_2_-C nanotubes.

### 3.3. Electrochemical Properties

**Figure 4:**
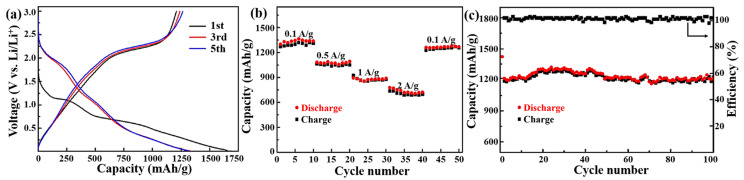
(**a**) Charge–discharge curves of VANS-MoS_2_-CNTs at 0.1 A/g; (**b**) rate performance of VANS-MoS_2_-CNTs at different current densities; (**c**) cyclic stability and Coulombic efficient of VANS-MoS_2_-CNTs at 0.1 A/g.

The as-obtained VANS-MoS_2_-CNTs with the advantages of a tubular structure, 2D MoS_2_ nanosheets, and sandwich-like carbon layers are expected to deliver high capacity and good rate capability as anodic material for LIBs, which can be confirmed by the following experiment results. Figure S4 displays the first and third cyclic voltammogram (CV) curves of the VANS-MoS_2_-CNT electrode at a scan rate of 0.2 mV/s. In the first cathodic sweep, the electrode shows two reduction peaks appearing at 0.48 and 0.89 V. The peak at 0.89 V is caused by the intercalation of Li^+^ ions in the crystal lattice of MoS_2_ with the conversion from 2H-MoS_2_ in 1T-Li_x_MoS_2_; furthermore, the other peak at 0.48 V can be assigned to the decomposition of Li_x_MoS_2_, leading to the generation of Li_2_S and Mo [54]. In the first charge process, the anodic peak at 1.76 V is due to the partial oxidation of Mo, and the other peak appearing at 2.32 V is assigned to the delithiation of Li_2_S [54,55]. In the third cycle, another two peaks located at 1.08 and 1.91 V emerge, which indicates the possible existence of a multistep Li^+^-insertion mechanism [55,56]. Figure 4a shows the charge–discharge curves of the VANS-MoS_2_-CNT electrode at 0.1 A/g. The initial discharge and charge capacities are 1587 and 1226 mAh/g, respectively. The decomposition of the electrolyte on the surfaces of the MoS_2_ nanosheets is responsible for the formation of a solid–electrolyte interface (SEI) layer in the first cycle, resulting in a low first-cycle columbic efficiency of 77.3% [57,58]. In the third and fifth charge–discharge curves, the VANS-MoS_2_-CNT electrode delivers discharge capacities of 1304 and 1293 mAh/g, respectively. To assess the rate capacity of VANS-MoS_2_-CNTs, the capacities at various current densities are shown in Figure 4b. As the current rates are 0.1, 0.5, 1, and 2 A/g, the average discharge capacities are 1330, 1070, 880, and 730 mAh/g, respectively. Furthermore, as the current rate returns to 0.1 A/g, the capacity rapidly increases to 1270 mAh/g, suggesting that the VANS-MoS_2_-CNT electrode has good reversible capability. Figure 4c indicates the cyclic stability and Coulombic efficiency of the VANS-MoS_2_-CNTs electrode. After 100 cycles, the electrode exhibits a high discharge capacity of 1270 mAh/g at 0.1 A/g. Furthermore, due to the hierarchical structure and carbon coating [58,59], the Coulombic efficiency of the VANS-MoS_2_-CNT electrode increases to over 98.5% in the following cycles.

**Figure 5:**
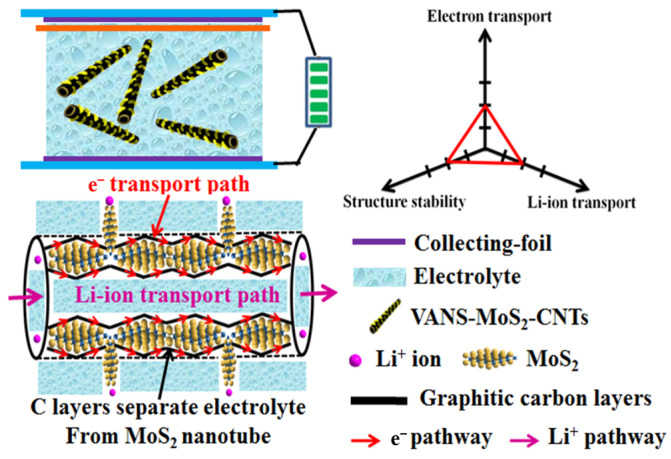
Schematic of LIBs with VANS-MoS_2_-CNTs, and the corresponding illustration of the Li^+^-ion insertion process.

The above results indicate that the VANS-MoS_2_-CNTs have excellent electrochemical performance. Moreover, compared to some other MoS_2_-based anodes [33,60,61,62,63,64,65], the VANS-MoS_2_-CNT electrode also exhibits good electrochemical performance (Table S1). From the perspective of “structure-performance”, the multilevel nanostructures of VANS-MoS_2_-CNTs provide advantages for enhanced performance. As illustrated in Figure 5, VANS-MoS_2_-CNTs are made up of C-MoS_2_-C nanotubes with VANS on the surface. Firstly, the nanotubular structure of VANS-MoS_2_-CNTs provides short and abundant transport paths for Li^+^ ions, improving Li^+^-ion diffusion [66]. Secondly, carbon layers cover the internal and external surfaces of the MoS_2_ nanotubes and a sandwich-like structure can be formed, which can effectively isolate the MoS_2_ nanotube from the electrolyte and therefore prevent the adverse reaction between MoS_2_ and the electrolyte. This advantage is very significant for enhancing the structural stability of MoS_2_. Thirdly, despite that fact that MoS_2_ nanotubes are partly separated from the electrolyte, VANS freely stand on the surfaces of MoS_2_ nanotubes, providing many active sites for Li^+^ and maintaining the advantage of 2D nanosheets. Fourthly, the sandwich structure of C-MoS_2_-C provides accessible pathways for electron transport [67]. This speculation can be proved by EIS test. Based on the EIS data (Figure S5) of VANS-MoS_2_-CNTs and MoS_2_ microspheres fabricated based on our previous work [40], VANS-MoS_2_-CNTs have lower charge transfer resistance than MoS_2_ microspheres (234.7 vs. 293.8 Ω), suggesting enhanced electron diffusion into and out of the VANS-MoS_2_-CNT electrode [68]. As expected, the VANS-MoS_2_-CNT electrode has excellent structural stability and good Li^+^/electron transport, leading to high capacity, insensible capacity fading, and good cyclic stability.

## 4. Conclusions

In conclusion, a simple, economical, and two-step method has been developed to fabricate 3D multilevel VANS-MoS_2_-CNTs. The as-obtained heterostructure shows the desired advantages of MoS_2_ nanosheets and sandwich-like nanotubes. This desirable structure exhibits high structural stability and good Li^+^/electron transport properties due to the carbon layers, nanotubular structure, and free-standing MoS_2_ nanosheets. Consequently, these massively prepared VANS-MoS_2_-CNTs have high discharge capacity and good cyclic performance. Thus, VANS-MoS_2_-CNTs have the potential to be employed in high-performance LIBs.

## Data Availability

Data sharing is not applicable to this article.

## Supplementary Materials

The following supporting information can be downloaded at: https://www.mdpi.com/article/10.3390/nano13142088/s1, Figure S1: (a) SEM and (b) TEM images of VANS-MoS_2_-NTs, and the inset of (a) is the high-magnified SEM image; Figure S2: N_2_ adsorption/desorption isotherms of VANS-MoS_2_-CNTs; Figure S3: SEM and EDS-mapping images of MoS_2_ nanotubes covered by carbon under the high concentration of glucose solution; Figure S4: CV curves for VANS-MoS_2_-CNTs at a scan rate of 0.2 mV/s; Figure S5: Nyquist plots of VANS-MoS_2_-CNTs and MoS_2_ nanoflowers; Table S1: The comparison of the electrochemical performance of VANS-MoS_2_-CNTs and some other previously reported MoS_2_-based anodes for LIBs (see Refs. [33,60,61,62,63,64,65]).
