# Supplementary material for: A 3D Multilevel Heterostructure Containing 2D Vertically Aligned MoS2 Nanosheets and 1D Sandwich C-MoS2-C Nanotubes to Enhance the Storage of Li+ Ions
Source: Nanomaterials (Basel). 2023 Jul 18;13(14):2088. doi: 10.3390/nano13142088 (PMC10384978; doi:10.3390/nano13142088)
Supplement: Supplementary file 1 [file nanomaterials-13-02088-s001.zip › nanomaterials-2497756-supplementary.pdf]

Supporting Information

A 3D Multilevel Heterostructure Containing 2D Vertically Aligned MoS_2 Nanosheets and 1D Sandwich C-MoS_2-C Nanotubes to Enhance the Storage of Li^+ Ions

Yiyang Zhao[1], Wenhao Luo[2], Huiqing Luo[1], Xiaodi Liu[1,*] and Wenjun Zheng[2,*]

[1]College of Chemistry and Pharmaceutical Engineering, Nanyang Normal University, Nanyang 473061, China

[2]Department of Chemistry, Key Laboratory of Advanced Energy Materials Chemistry (MOE), College of Chemistry, Nankai University, Tianjin 300071, China

*Correspondence: 20122029@nynu.edu.cn; zhwj@nankai.edu.cn

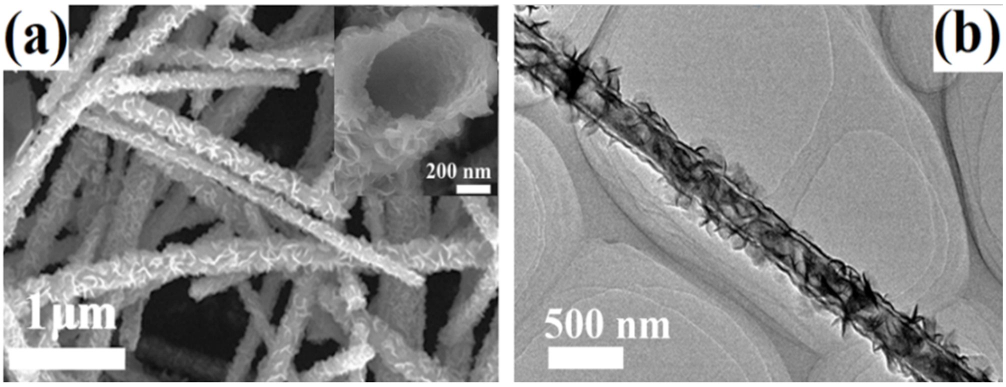

Figure S1 (a) SEM and (b) TEM images of VANS-MoS_2-NTs, and the inset of (a) is the high-magnified SEM image.

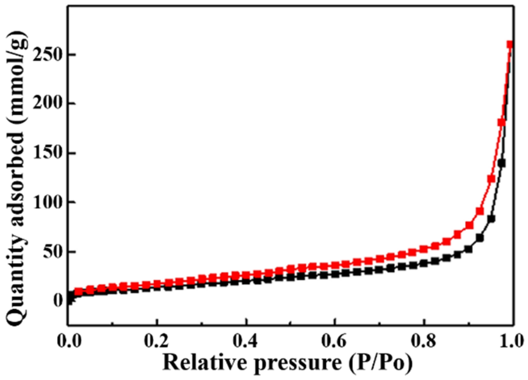

Figure S2 N_2 adsorption/desorption isotherms of VANS-MoS_2-CNTs.

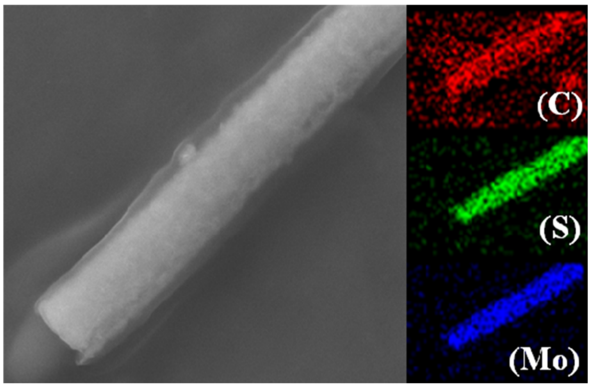

Figure S3 SEM and EDS-mapping images of MoS_2 nanotubes covered by carbon under the high concentration of glucose solution.

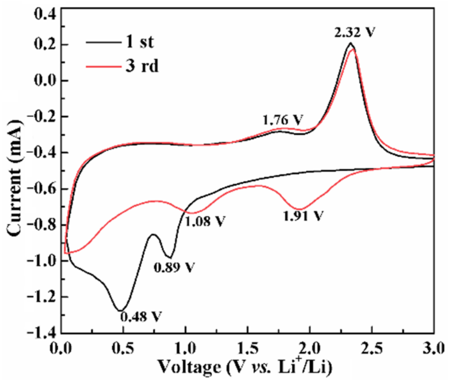

Figure S4 CV curves for VANS-MoS_2-CNTs at a scan rate of 0.2 mV/s.

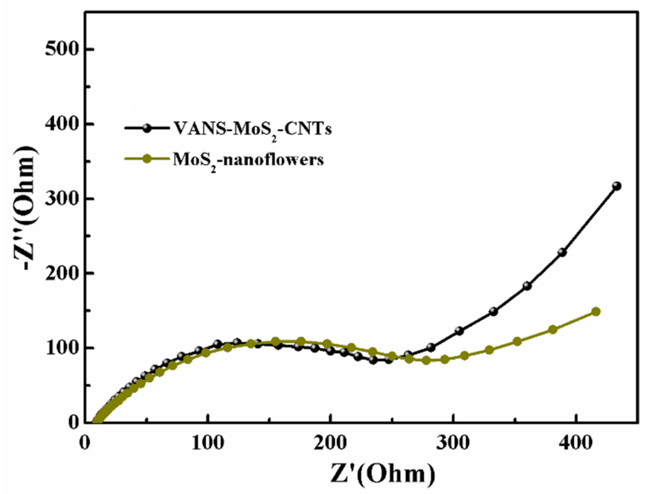

Figure S5 Nyquist plots of VANS-MoS_2-CNTs and MoS_2 nanoflowers.

Table S1. The comparison of the electrochemical performance of VANS-MoS_2-CNTs and some other previously reported MoS_2-based anodes for LIBs.

**Samples**	**Cycling stability mAh/g-cycles-A/g**	**Rate capability mAh/g-A/g**	**Ref.**
VANS-MoS_2-CNTs	1270-100-0.1	730-2	In this work
C@MoS_2 nanoboxes	952/200/0.4	689/2	[1]
Carbon nanotube hybrids with MoC and MoS_2	1200/200/0.1	680/1	[2]
Hollow microsphere@solid nanosphere MoS_2	302/100/0.1	*ca*. 260/1	[3]
Graphene quantum doped MoS_2 nanosheets	1031/80/0.1	660/5	[4]
MoS_2/N-graphene nanocomposites	820/100/1	700/2	[5]
N-doped graphene/MoS_2/N-doped graphene heterostructure	552/600/1	528/2	[6]
MoS_2-rGO/hollow carbon spheres network	1145/100/0.1	753/2	[7]

[1] Yu, X.Y.; Hu, H.; Wang, Y.W.; Chen, H.Y.; Lou, X.W. Ultrathin MoS_2 nanosheets supported on N-doped carbon nanoboxes with enhanced lithium storage and electrocatalytic properties. *Angew. Chem. Int. Ed.* **2015**, *54*, 7395-7398.

[2] Li, X.; Zhang, J.Y.; Wang, R.; Huang, H.Y.; Xie, C.; Li, Z.H.; Li, J.; Niu, C.M. In situ synthesis of carbon nanotube hybrids with alternate MoC and MoS_2 to enhance the electrochemical activities of MoS_2, *Nano Lett*. **2015**, *15*, 5268-5272.

[3] Guo, B.; Yu, K.; Song, H.; Li, H.; Tan, Y.; Fu, H.; Li, C.; Lei, X.; Zhu, Z. Preparation of hollow microsphere@onion-like solid nanosphere MoS_2 coated by a carbon shell as a stable anode for optimized lithium storage. *Nanoscale* **2016**, *8*, 420-430.

[4] Guo, J.; Zhu, H.; Sun, Y.; Tang L.; Zhang, X. Boosting the lithium storage performance of MoS_2 with graphene quantum dots. *J. Mater. Chem. A* **2016**, *4*, 4783-4789.

[5] Zhao, C.; Wang, X.; Kong, J.; Ang, J.M.; Lee, P.S.; Liu, Z.; Lu, X. Self-assembly-induced alternately stacked single-layer MoS_2 and N-doped graphene: A novel van der Waals heterostructure for lithium-ion batteries. *ACS Appl. Mater. Interfaces* **2016**, *8*, 2372-2379.

[6] Chen, B.; Meng, Y.H.; He, F.; Liu, E.Z.; Shi, C.S.; He, C.N.; Ma, L.Y.; Li, Q.Y.; Li, J.J.; Zhao, N.Q. Thermal decomposition-reduced layer-by-layer nitrogen-doped graphene/MoS_2/nitrogen-doped graphene heterostructure for promising lithium-ion batteries. *Nano Energy* **2017**, *41*, 154-163.

[7] Hu, X.; Li, Y.; Zeng, G.; Jia, J.C.; Zhan, H.B.; Wen, Z.H. Three-dimensional network architecture with hybrid nanocarbon composites supporting few-layer MoS_2 for lithium and sodium storage. *ACS Nano* **2018**, *12*, 1592-1602.
